# Thermal Plasticity in Life-History Traits in the Polymorphic Blue-Tailed Damselfly, *Ischnura elegans*: No Differences between Female Morphs

**DOI:** 10.1673/031.011.11201

**Published:** 2011-08-29

**Authors:** Niels Bouton, Arne Iserbyt, Hans Van Gossum

**Affiliations:** Evolutionary Ecology Group, Department of Biology, University of Antwerp, Groenenborgerlaan 171, B-2020, Antwerp, Belgium

**Keywords:** adaptation, egg mortality, hatching time, phenotypic plasticity, temperature

## Abstract

Female polymorphism is observed in various animal species, but is particularly common in damselflies. The maintenance of this polymorphism has traditionally been explained from frequency and density dependent sexual conflict, however, the role of abiotic factors has recently attracted more interest. Here, the role of ambient temperature in shaping life-history was investigated for the three female morphs of *Ischnura elegans* (Vander Linden) (Zygoptera: Coenagrionidae). Eggs were obtained from the three mature female morphs for two populations in the Netherlands. Using a split-brood design, eggs of both populations were divided between a cold and a warm treatment group in the laboratory, and egg survival and hatching time were measured. Significant thermal plasticity was found in both hatching time and egg survival between both temperature treatments. However, individuals born to mothers belonging to different colour morphs did not differ in their response to temperature treatment. Independent of colour morph, clear differences in both life-history traits between the populations were found, suggesting local adaptation. Specifically, individuals from one population hatched faster but had lower egg survival in both thermal regimes. The selection force establishing fast hatching could be (facultative) bivoltinism in one of the populations compared to univoltinism in the other. This would be in line with the more southern (and more coastal) location of the presumed bivoltine population and the inverse relation between voltinism and latitude known from earlier studies. However, other natural selection forces, e.g. deterioration of the aquatic habitat, may also drive fast hatching.

## Introduction

Phenotypic plasticity induced by temperature (thermal plasticity) has been described for many organisms (Angiletta 2009). Usually thermal plasticity is presented as a reaction norm, describing the relationship between temperature and a continuous phenotypic variable (Angiletta 2009). Thermal reaction norms themselves can be under natural selection. This process may lead to thermal adaptation in populations that live under different temperature conditions, for instance at different altitudes or latitudes (Angiletta 2009). Thermal plasticity and adaptation are often reported in ectotherms because these organisms are largely dependent on external heat for any activity. Life-history parameters, such as development time, can be both plastic and locally-adapted (e.g., James and Partridge 1995; Liefting et al. 2009).

Thermal plasticity can also be studied in relation to polymorphism. After all, if species are polymorphic, morphs may perform optimally at different temperatures (Ahnesjö and Forsman 2003). Female polymorphisms in coenagrionid damselflies (Odonata: Zygoptera) have mostly been explained from frequency and/or density dependent sexual conflict (Van Gossum et al. 2008; Gosden and Svensson 2009). The adult terrestrial stage of damselflies is usually short-lived, and in many species the operational sex ratio is malebiased, leading to competition among males for access to females (Corbet 1999). As a consequence, females typically have many more mating possibilities than required for optimising reproductive success (Arnqvist and Nilsson 2000). Therefore, females regularly have to fend off mating attempts, and mating often involves male coercion. The existence of female colour morphs, one of which (the andromorph) resembles the conspecific male in phenotype, is hypothesized to be instrumental in reducing male harassment (Andrés et al. 2002; Van Gossum and Sheratt 2008; Van Gossum et al. 2008). Indeed, it has been suggested that this male-like female morph deceives males in that males do not recognise andromorphs as females easily (e.g., Robertson 1985; Cordero et al, 1998; Sherratt and Forbes, 2001). In addition to sexual conflict, differences in life-history traits between morphs may affect female polymorphism in damselflies. For instance, development time of larvae differed between female morphs of the damselfly *Ischnura elegans* (Abbott and Svensson 2005). Furthermore, female morphs of the damselfly *I. senegalensis* were found to have morph-specific fecundity and egg size (Takahashi and Watanabe 2010a). Such differences in life-history traits could result in natural selection acting on the morphs and influencing morph frequencies (Abbott and Svensson 2005).

A natural selection force that potentially affects morphs of damselflies differentially is ambient temperature. For example, when comparing female morph frequencies of populations of the blue-tailed damselfly, I. *elegans* across the Netherlands, andromorph frequency appeared to correlate negatively with ambient temperature (Hammers and Van Gossum 2008; see also Iserbyt et al. 2009). Causes for these types of correlations often remain poorly understood (but see Cooper 2010). In temperate climates colour morphs that heat up faster may have a selective edge, while the morph-related ability to deal with negative effects of solar radiation may be favoured in the tropics (c.f. Cooper 2010). In temperate wild-caught blue damselflies, *Enallagma cyathigerum*, protein content correlated positively with ambient temperature for both adult female morphs, but the effect was more pronounced for andromorphs (Bots et al. 2009). However, no differences between morphs were observed in field body temperature or in heating rate under controlled laboratory conditions (Bots et al. 2008). Also, no morph-specific differences in behaviour of *Nehalennia irene* were found with respect to temperature (Iserbyt and Van Gossum 2009). Clearly, more experimental studies are needed to clarify whether lifehistory traits are differentially shaped by ambient temperature for different maternal morphs in female polymorphic insects. Such studies should not limit themselves to the adult stage but should also focus on egg or larval stages. Indeed, reproductive output and contributions to future generations are shaped by the entire development from egg to adult and by internal and external factors affecting these life-history stages.

Here, we investigate temperature effects on the egg stage for the three different maternal morphs of *Ischnura elegans* (Vander Linden) (Zygoptera: Coenagrionidae) in two populations in the Netherlands. Following a split-brood design, egg clutches of different female morphs were divided between a warm and a cold climate room. Egg survival and hatching time were measured for both temperature regimes. Thermal plasticity would manifest itself as different responses of the eggs to these temperature regimes. Typically, ectotherms are expected to perform better at the higher temperature. Maternal morphs may influence the response values that are measured. Finally, local adaptation may play a role in the experiment, since gene flow between populations may be restricted due to the patchy distribution of freshwater bodies suitable for *I. elegans*.

## Methods

### Study species

The blue-tailed damselfly, *I. elegans*, is common in mesotrophic to eutrophic wet habitats throughout Eurasia. Adults emerge in spring and summer (May–August) and are short-lived, but estimates of daily survival vary between studies: 0.812 ± 0.031 (mean ± SE) for males and 0.579 ± 0.086 for females (Anholt et al. 2001) as compared to 0.960 ± 0.118 for males and 0.898 ± 0.093 for females (Cordero et al. 1998). Males and females forage and mate in the shoreline vegetation. Mating takes several hours. Females store sperm, and fertilization occurs just prior to egg-laying. Egg-laying is done in submerged vegetation in the late afternoon. Eggs hatch a few weeks after oviposition. In northern Europe, larvae overwinter and normally complete their life cycle the next spring or summer, referred to as univoltinism. Bivoltinism, a second cycle in the same year, or even multivoltinism occurs further south (Corbet et al. 2006; Dijkstra and Lewington 2006).

*I. elegans* displays three discrete, genetically encoded female colour morphs the andromorph and two heteromorphs (often referred to as ‘gynomorphs’). The andromorph mimics the male blue-tailed damselfly, as it has the same blue and black coloration and black patterning as the male. The other two morphs, *infuscans* and *rufescens-obsoleta*, are together referred to as heteromorphs. *Infuscans* has olivegreen/brown and black colouration with black patterning similar to males and andromorphs. *Rufescens-obsoleta* has olive-brown/brown and black patterning and is unique in the reduction or absence of two of the black stripes on the thorax. In addition, each of these morphs is preceded by ontogenetic color changes before the described adult phenotypes are reached. The three alleles determining the color morphs are located on a single autosomal locus, have a simple dominance hierarchy (A > I > RO), and have a phenotypic expression that is limited to the females (Sánchez-Guillén et al. 2005). Given this dominance hierarchy and the latency of expression in males each female type can produce a mixture of morphs in her offspring.

### Source populations

Two populations in the Netherlands were selected for the thermal plasticity experiment in a straight line 133 km apart. One site is situated near Koudekerke (51° 29′ 09Z″ N, 3° 32′ 25″ E) at 2 km from a sea arm in the southwest of the Netherlands. The other site is situated near Loosdrecht (52° 12′ 58″ N, 5° 04′ 48″ E) in the Loosdrecht Lakes area in the centre of the Netherlands, at 41 km from the coast. Both sites are near weather stations, Vlissingen (5 km from Koudekerke) and De Bilt (12 km from Loosdrecht), from which we obtained 30-year climate data. Average temperatures for winter, spring, summer, and autumn are 4.2, 8.9, 16.8, and 11.6° C at Koudekerke and 3.3, 8.9, 16.6, and 10.2° C at Loosdrecht. Thus, ambient temperatures are roughly the same, but autumn temperatures are 1.4° C higher at Koudekerke.

Morph frequencies for both populations were estimated in the summer of 2009 prior to collecting individuals (see below) on June 24 in Koudekerke and July 13 in Loosdrecht. This was done by walking slowly along the shoreline sweeping a 40 cm diameter insect net through the vegetation in a figure eight (Hammers and Van Gossum 2008). During these sweep net samples, time was recorded and the effort was continued until at least 30 adult females were caught. This allowed for an estimation of density (individuals/second) and female morph frequency. Koudekerke had a high andromorph frequency (71%), with frequencies of the heteromorphs being 13% *infuscans* and 16% *rufescens-obsoleta*. Density was found to be very high (0.45 individuals/s) as compared to other sites in the Netherlands (cf. Hammers and Van Gossum 2008). Loosdrecht had an intermediate andromorph frequency (50%), with frequencies of the heteromorphs being 33% *infuscans* and 17% *rufescens-obsoleta*. Density was also high at 0.33 individuals/s.

### Experimental set-up

We aimed at using egg clutches of 20 individuals of each female morph of both locations (60 for each site; total 120). Females were caught between 11:00 and 16:00, before they would normally start laying eggs (i.e., in late afternoon). Females were placed in transparent square plastic cups (L × W times; H: 9 × 6 × 10 cm). These were closed with mesh and rubber band, furnished with wet filter paper for egg laying and a branch for resting. A total of 123 females were caught at Koudekerke (on 28 July and 3 August 2009) and 96 at Loosdrecht (on 31 July, and the 5 and 11 August 2009) to obtain sufficient egg clutches. Plastic cups, with females, were transported to the laboratory. After 24 hours, females were removed from the cups and filter papers with the eggs were placed in Petri dishes of 15 cm diameter with dechlorinated tap water. Eventually, the numbers of egg clutches were as follows: Koudekerke, andromorphs, N= 19, *infuscans*, N= 20, *rufescens-obsoleta*, N= 17; Loosdrecht, andromorphs, N= 18, *infuscans*, N= 20, *rufescens-obsoleta*, N= 17. Note that a few incomplete clutches (< 50 eggs) were used to boost replication number.

A split-brood design was used: the filter paper was divided in two parts with approximately half of the eggs each and put in two Petri dishes. Eggs were counted, and the Petri dishes were placed in two temperature-controlled rooms. Temperatures in the rooms were registered every half hour on a data logger (Easy Log USB 4.5, www.easylogusb.software.informer.com). Temperatures were chosen within the range of spring/summer temperatures in the Netherlands, representing realistic colder and warmer circumstances or sites. The ‘cold’ room was kept at 17.1 ± 0.05° C, the ‘warm’ room at 20.7 ± 0.03° C, while in both rooms a light-dark regime was held constant (16:8 L:D). This light regime was chosen to mimic the natural situation in the Netherlands for August 15. Also by keeping photoperiod constant, we were able to fully investigate the role of temperature on offspring fitness, as this is the primary aim of this study. Hatching was scored on a daily basis, until all clutches had produced larvae. Usually the vast majority of eggs hatched on a single day. Hatching time and egg survival were defined, respectively, as the median number of days that it took for eggs to hatch and the proportion of eggs that eventually hatched (in a given Petri dish).

### Statistics

All analyses were performed in SAS 9.2 (SAS Institute Inc., Cary, NC, USA, www.sas.com). Since the average clutch size for *I. elegans* is around 250 eggs (Svensson and Abbott, 2005), clutches of less than 50 eggs were considered incomplete and therefore omitted from the fecundity analysis. Differences in fecundity between female morphs were analysed using an ANOVA (‘proc mixed’ in SAS), including site as a random variable. A similar model was performed with hatching time as the dependent variable but with temperature treatment and the morph*treatment interaction as additional explanatory variables. Finally, a general linear model (‘proc genmod’ in SAS) was used with egg survival (proportion of eggs hatched) as the dependent variable and the same explanatory variables as in the model for hatching time. Since each clutch was split in two and reared separately, we controlled for dependence of the maternal individual by adding an individual based repeated statement in the analyses for the reaction norm experiment.

### Results

**Figure 1.**
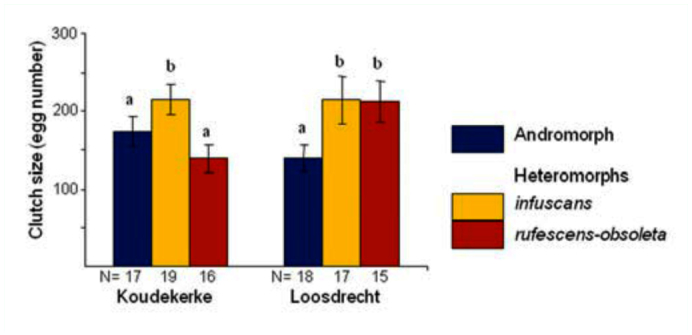
Number of eggs laid (mean ± 1 SE) by each female morph for both populations. The different letters on top of each histogram indicate significant differences (P < 0.05) of pairwise post hoc comparisons for each population. High quality figures are available online.

When controlling for potential variation among sites, clutch size differed between morphs (F_2,102_ = 3.84; p = 0.025). Specifically, in Koudekerke, andromorphs had an equal number of eggs oviposited as *rufescensobsoleta* females (t = 1.09; P = 0.28), and both of these values were significantly lower than *infuscans* females (both < 0.05; Figure 1). In Loosdrecht, only andromorph females had a lower fecundity relative to the others, *infuscans* (t = -2.38; P = 0.019) and *rufescensobsoleta* (t = -2.25; P = 0.027).

**Figure 2.**
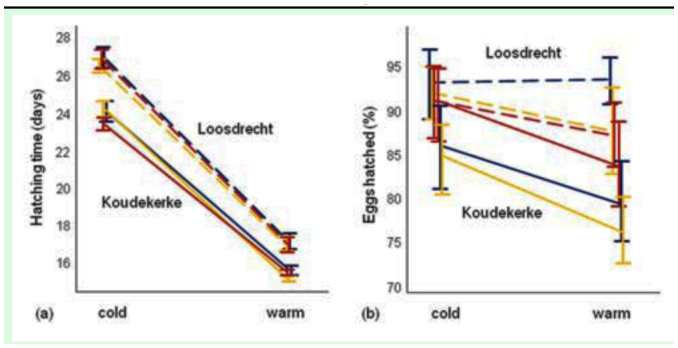
Reaction norms of hatching time (a) and egg survival (b) for the three female morphs of the Koudekerke (solid lines) and Loosdrecht (dashed lines) population when reared under controlled warm and cold laboratory conditions (mean ± 1 SE). The same colours are used as in Figure 1: blue = andromorph, yellow = *infuscans*, and red = *rufescens-obsoleta*. High quality figures are available online.

There was no effect of maternal morph on hatching time (F_2,218_= 0.75; P = 0.47), and this was irrespective of temperature treatment (treatment*morph: F_2,216_= 0.42; P = 0.66; Figure 2A). Hatching time differed between the warm and cold treatment (F_1,220_= 1618.2, p< 0.0001), with hatching in the warm treatment being almost twice as fast as in the cold treatment. When specifically looking at the effect of study site and treating this variable as fixed, it became clear that clutches from the Koudekerke population hatched faster than those from the Loosdrecht population (F_1,135_= 151.5, p< 0.0001). Moreover, there was a significant treatment*site interaction (F_1,135_= 7.21, p= 0.008), specifically meaning that eggs from Koudekerke hatched approximately two days earlier in the warm treatment, compared with three days earlier in the cold treatment.

Egg survival also depended on temperature treatment (*x*21= 4.93, p= 0.026), with highest survival in the cold treatment compared with the warm treatment (0.88 ± 0.02 vs. 0.84 ± 0.02, respectively; Figure 2B). As with hatching time, we observed no differences between maternal morphs (*X*22= 0.98, p = 0.61). However, we again observed strong differences between sites (X^2^1= 9.27, p= 0.002), with the Koudekerke population having the lowest egg survival (0.83 ± 0.02) compared with the Loosdrecht population (0.90 ± 0.02).

## Discussion

### Clutch sizes

Clutch sizes differed between female morphs at both sites (Figure 1). These findings were roughly similar to those reported elsewhere for *I. elegans*, i.e., smaller clutches in andromorph females (Abbott and Svensson 2005). Possibly, this reflects a cost of resembling the male. Males have a narrower abdomen, and by mimicking this character andromorphs may have less space to store eggs (Abbott and Svensson 2008). In this study, the cause of smaller clutch sizes of *rufescens-obsoleta* at Koudekerke was not investigated. However, variation in clutch size of heteromorph females in *I. elegans* has also been observed elsewhere (Gosden and Svensson 2009) and is explained by the learned mate-recognition hypothesis. The hypothesis states that common morphs are more often recognized by males as potential mates than rare morphs and therefore experience more male harassment, resulting in less foraging time and smaller clutches (Takahashi and Watanabe, 2010b; Gosden and Svensson 2009). This may play a role in our study, but data on male harassment that could lend further support are lacking. In addition, this hypothesis is better tested in a large number of populations instead of just two (see Gosden and Svensson 2009).

It should also be noted that the size of a single clutch does not necessarily reflect lifetime reproductive success (Bots et al. 2010). Factors such as interclutch interval (Forsman 2001) and longevity (Sirot and Brockmann 2001) should also be taken into consideration. In the damselfly *Coenagrion puella* most variation in lifetime reproductive success of females in the wild arose from longevity (70%) rather than interclutch interval (20%) or clutch size (10%) (Banks and Thompson 1987). A similar pattern was found in captivity for *I. graellsii*, a sister species of *I. elegans* (Cordero 1991).

### Phenotypic plasticity

Temperature-induced phenotypic plasticity was most pronounced in hatching time. Hatching in the cold treatment took circa nine days longer on average than in the warm treatment, which means an almost twofold increase and a rather steep reaction norm given the small difference in temperature (ca. 3.6° C). Although thermal plasticity is not uncommon in ectotherms (e.g. James and Partridge 1995; Liefting et al. 2009), such steep reaction norms may be typical for insect orders that originated in tropical environments, such as Odonata (Pritchard et al. 1995). Being adapted to warm areas, Odonata usually survive cold periods in the larval stage in diapause. For most species, the optimal temperature for development is above 20° C, and no growth is observed below 10° C (Pritchard et al. 1995; Corbet 1999). This relatively poor adaptation to colder climates may explain the steep reaction norm we observed and agrees with earlier findings in damselflies (Van Doorslaer and Stoks 2005a, b). While hatching time differed greatly, a much smaller, though significant, difference in egg survival between temperature treatments was found. Eggs survived slightly better in the cold treatment. This may indicate a trade-off between hatching time and egg survival. The fact that the differences in egg survival were rather small might be an effect of canalization of egg survival against temperature fluctuations (Liefting et al. 2009), at least for the temperatures used in the experiment.

### Local adaptation

Interestingly, divergent responses at the population level were found in both hatching time and egg survival. The (Koudekerke) population whose eggs hatched faster experienced at the same time a lower egg survival, and this finding was the same for both laboratory temperature treatments (Figure 2). This again suggests a trade-off between the two characters: fast hatching would occur at the expense of the proportion of eggs surviving. It also suggests a heritable component in these life-history traits and may therefore be interpreted as local adaptation. Given the differences between populations in both hatching time and egg survival, the nearly parallel reaction norms, and the similar summer temperatures at the two sites, it can be deduced from our data that temperature is not the key selection pressure driving the process. Non-quantitative (casual) observations point at an alternative explanation: bivoltinism at Koudekerke vs. univoltinism at Loosdrecht. From the end of July to the beginning of August, when females were collected for the experiment, there were clear differences in population structure between both populations. The Koudekerke population was still at high density with many tenerals (i.e. freshly hatched subadult individuals), while the Loosdrecht population was in decline with a diminishing number of adults and very few tenerals. Thus, it is possible that the Koudekerke population stood at the beginning of a second generation in the same season, while the Loosdrecht population was ending the only generation of that year. It is known that the widespread *I. elegans* shows a pronounced inversed relation between latitude and voltinism (Corbet et al. 2006). *I. elegans* has been listed as bivoltine in Belgium (directly south of The Netherlands) and northern France, and mainly univoltine in The Netherlands (Corbet et al. 2006). The coastal population we used is near the Belgium border, and the maritime climate in the estuarine area may allow for a slightly prolonged growing season as compared to the inland population and hence bivoltinism. It is generally assumed that voltinism can influence development time, but no difference in egg hatching time was observed between univoltine and semivoltine European populations of the damselfly *E. cyathigerum* (De Block et al. 2008). Note, in this respect, that the two to three day difference in egg hatching time that we observed may reflect a much larger difference in total development time (including the larval stage).

Voltinism may be an explanation for the observed pattern, but many alternative circumstances have been described that strongly favour fast egg hatching in insects. For instance, in *Drosophila melanogaster* rapid development increases fitness by a positive effect on larval survival (James and Partridge 1995). This is caused by deterioration of the breeding substrate (usually a single fruit) over time as a result of, among others factors, crowding and nutrient depletion (James and Partridge 1995). In damselflies time constraints imposed by seasonality (Johansson and Rowe 1999) or desiccation of temporary ponds (De Block et al. 2005) can drive fast development. The high tendency of cannibalism in many Odonata may compound the effect (Johansson et al. 2001). We have not studied any of these potential selection pressures in our study populations, but such circumstances may help to explain the pattern observed.

### Maternal morph, temperature and development

Temperature influenced egg survival and hatching time in almost exactly the same way for all three maternal morphs. More importantly, egg development was not found to vary between maternal morphs in the first place. This contrasts with the finding that development time (including the larval stages) of *I. elegans* was found to be different between morphs in a population from Sweden (Abbott and Svensson 2005). Development of offspring of *rufescens-obsoleta* took about 14 days less than that of the andromorph and *infuscans* (ca. 280 vs. 294 days, Abbott and Svensson 2005). However, some *rufescensobsoleta* females were collected from nearby populations (Abbott and Svensson 2005), and this may have contributed to the observed differences in development time, especially in light of the interpopulational differences reported here. Like our study, other studies found no differences in development time of offspring between maternal morphs of *I. graellsii* (Cordero 1994) and *E. cyathigerum* (Bots et al. 2010). However, it remains conceivable that maternal morph effects on development time for the Dutch populations are not yet detectable in egg hatching time, since this comprises only about one tenth of the total development time. More studies on both the population and species level are needed to reach firm conclusions about maternal morph effects on development time and interpopulational variation.

### Conclusion

A strong plastic response to temperature was found in hatching time of *I. elegans* eggs. A much smaller response was found in egg survival, suggesting canalization against temperature differences of this magnitude. Local adaptation was found in both hatching and egg survival. The eggs of females from a southern coastal population hatched faster but had lower survival, suggesting a trade-off between both traits. A relatively high percentage of eggs from females of the northern inland population hatched but somewhat slower. The evolution of fast (egg) development in the coastal population may be driven by the occurrence of a second generation in the summer season (bivoltinism), but other explanations cannot be excluded at this time.
